# Alveolar antral artery: cone beam computed tomography study and clinical context

**DOI:** 10.7717/peerj.16439

**Published:** 2023-11-30

**Authors:** Ján Staněk, Kateřina Machálková, Magdalena Staňková, Jana Zapletalová, Tat’ána Kocurová

**Affiliations:** 1 Institute of Dentistry and Oral Science, Faculty of Medicine and Dentistry, Palacký University Olomouc, Olomouc, Czech Republic; 2 Department of Medical Biophysics, Faculty of Medicine and Dentistry, Palacký University Olomouc, Olomouc, Czech Republic

**Keywords:** Alveolar-antral artery, Lateral sinus lift complication, cbct study, Dental implants perioperative complications, Sinus floor augmentation

## Abstract

**Purpose:**

Anastomosis between posterior superior alveolar artery and infraorbital artery can go through bony canal in the lateral wall of the maxilla. This artery is called alveolar antral artery. It can complicate lateral sinus lift procedure by bleeding and hemosinus formation or bone graft wash out. The artery can also go in soft tissues where is not visible on cone beam computed tomography. In previous studies, the relation of this artery to sinus floor or alveolar process was measured. These structures are highly unstable during lifetime and after tooth loss. The aim of this study is to study presence and relations of bony canal in the lateral maxillary wall, to characterize the group of patients which is more likely to have bone canal in the lateral maxilla. The aim and the novelty of this study is the describing of the relationship of the bony canal to the more stable structure of hard palate and describing the relation of presence of bony canal on width of maxillary sinus, and to facilitate the prediction of presence of the alveolar antral artery.

**Materials:**

The cone beam computed tomography scans of the patients (251 in number) of the university hospital were examined for presence of alveolar antral artery (148 was fulfilled inclusion criteria), patient were characterized by gender, age, and sinus type (wide, average, narrow). The diameter of the bony canal and its relation to the level of sinus floor and hard palate were measured.

**Results:**

The cone beam computed tomography scans of 148 patients, out of it 55 man (37,2%) and 93 women (62,8%). Bony canal containing alveolar anastomosis was found in 69 cases (57,0%). Presence of the bony canal in the lateral wall of maxillae showed statistical probability depending on age with *p* = 0, 064 according to Mann-Whitney test. The older patients have more likely the bony canal. The presence of the alveolar antral artery was found more likely in the wide sinuses. The hard palate level can serve as a prediction point of alveolar antral artery only in first molar and second premolar region. In accordance with previous studies the width of bony canal is significantly higher in group of man (*p* = 0, 015). There was found a correlation between smaller distance of bony canal from sinus floor in the presence of teeth (*p* = 0, 067). After tooth loss the distance between sinus floor and bony canal increases, but the distance of bony canal to hard palate level stays constant. This can be explained hypothetically so that periodontal ligaments and root surface acts as a barrier for sinus pneumatization.

**Conclusion:**

Lateral sinus lifting in some cases can be unenviable, the knowledge about alveolar antral artery anatomy can reduce the risk of arterial bleeding. The cone-beam computed tomography is a routine examination prior to augmentation surgery and therefore the data obtained from it has an impact on clinical practice.

## Introduction

The alveolar antral artery (AAA) is an anastomosis between the posterior superior alveolar artery and the infraorbital artery. AAA provides blood supply to the maxillary sinus mucous membrane and maxillary periosteum. AAA could be injured during dentoalveolar and otorhinolaryngologic surgery such as lateral sinus lifting, Le Fort I fracture repair, orthognathic surgery or Caldwell Luc surgery (Taschieri & Rosano, 2010; Ella et al., 2008). AAA was described in 1934 (Strong, 1934).

Since 1986 when Tatum Jr (1986) described lateral sinus, this technique become predictable and routine. AAA presents a possible complication of the surgery. AAA injury can lead to graft wash out, hemosinus, hematoma and its infection, thus jeopardizing the outcomes of surgical time and cost demanding surgery (Varela-Centelles et al., 2020). A recent review showed that AAA was found in 100% in nine cadaver studies. CBCT studies found AAA between 32% and 93% (Sedov et al., 2020).

The alveolar antral artery was studied in a cadaver study where on 18 cadavers it was found in each cadaver. Intraosseous form was found in 56% (Traxler et al., 1999). The alveolar antral artery is constantly present and in literature can be also found as posterior superior alveolar artery (Park, Choi & Kim, 2012; Alves et al., 2023).

**Figure 1:**
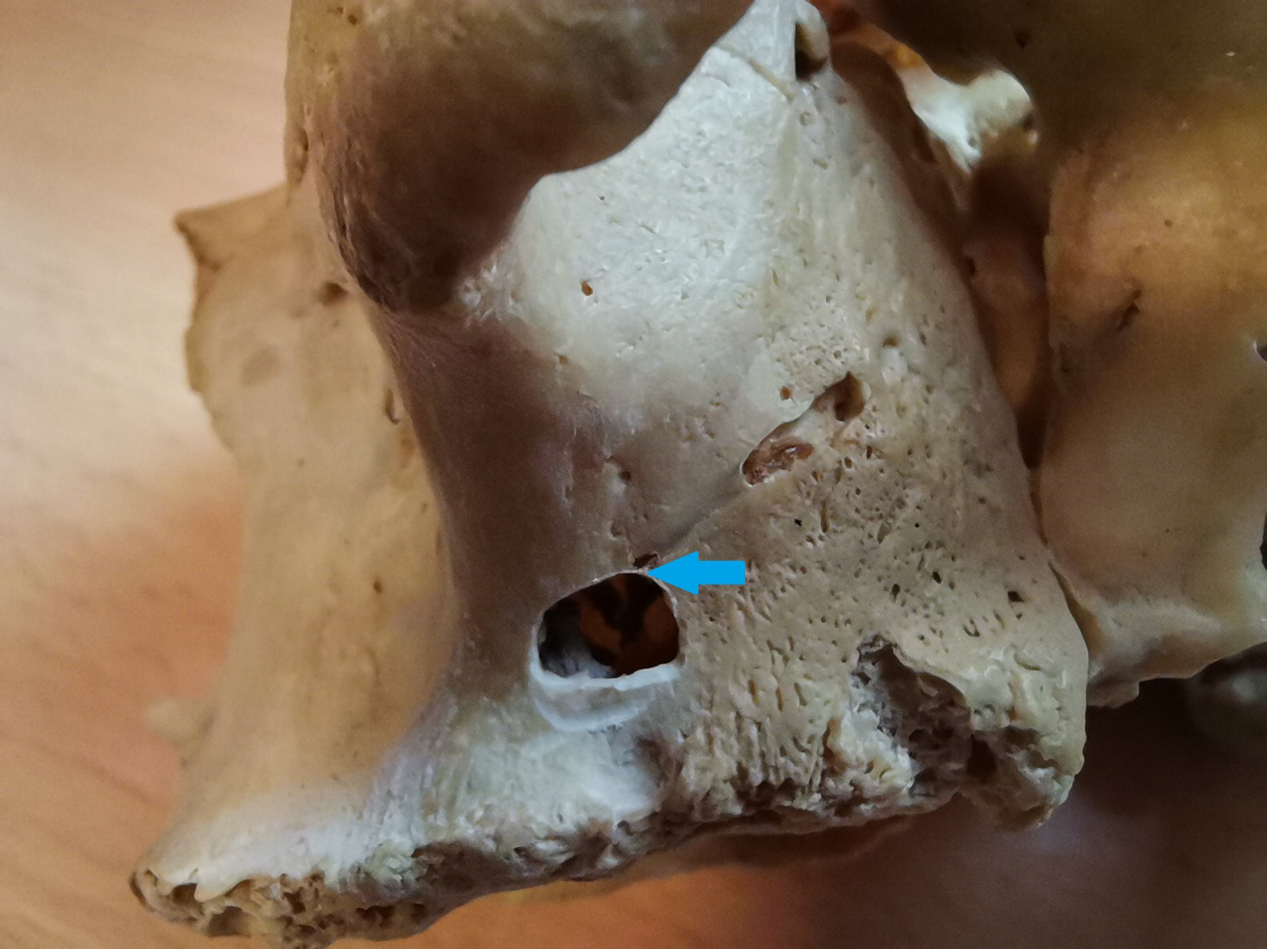
The alveolar antral artery on skull, the artificial lateral sinus lift window was drilled to show the possibility of the vascular injury. The collision of bony canal and lateral window osteotomy is marked by a blue arrow.

**Figure 2:**
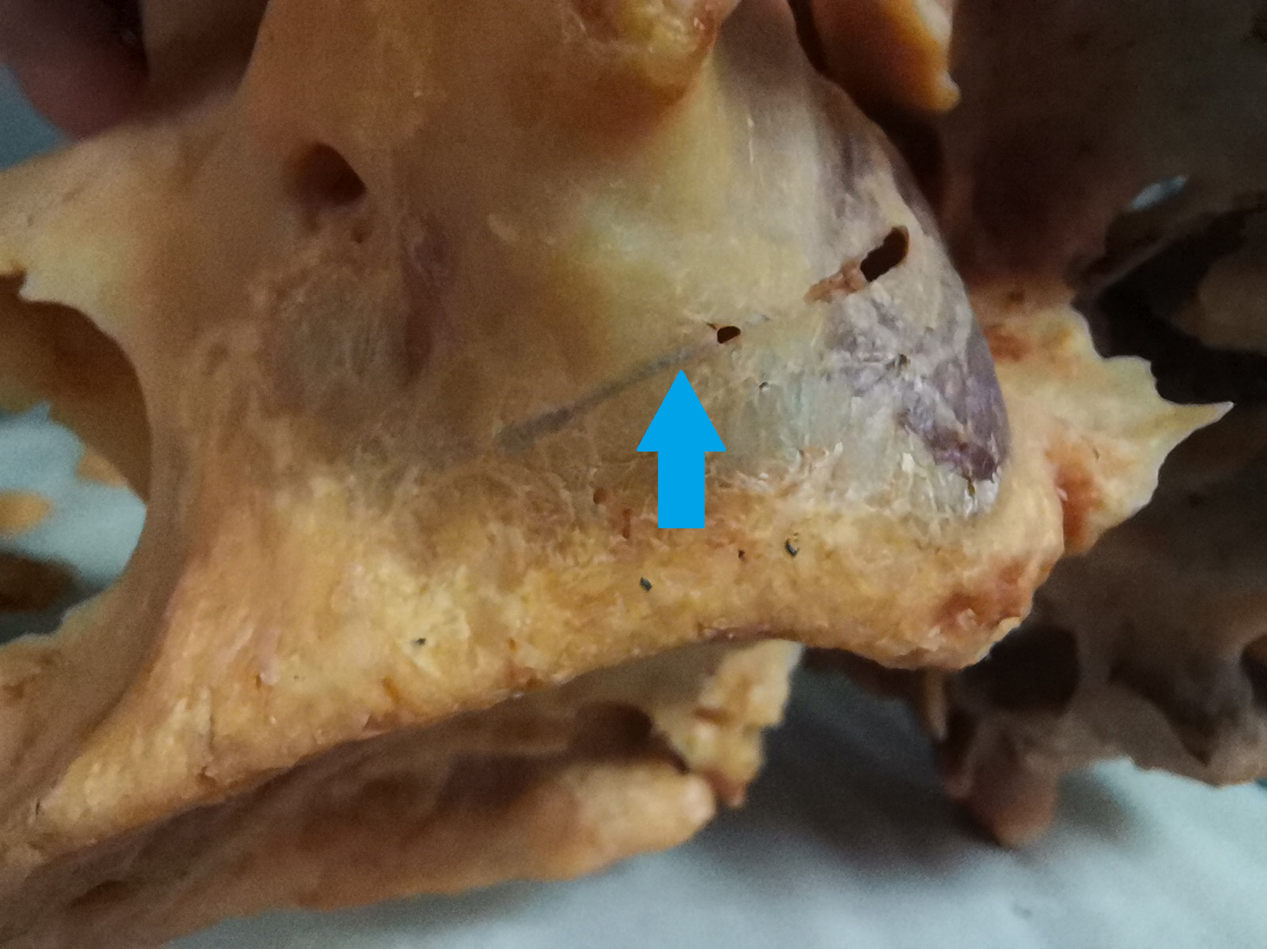
The bony canal of the alveolar antral artery on the skull. The bony canal is visible and marked by a blue arrow.

**Figure 3:**
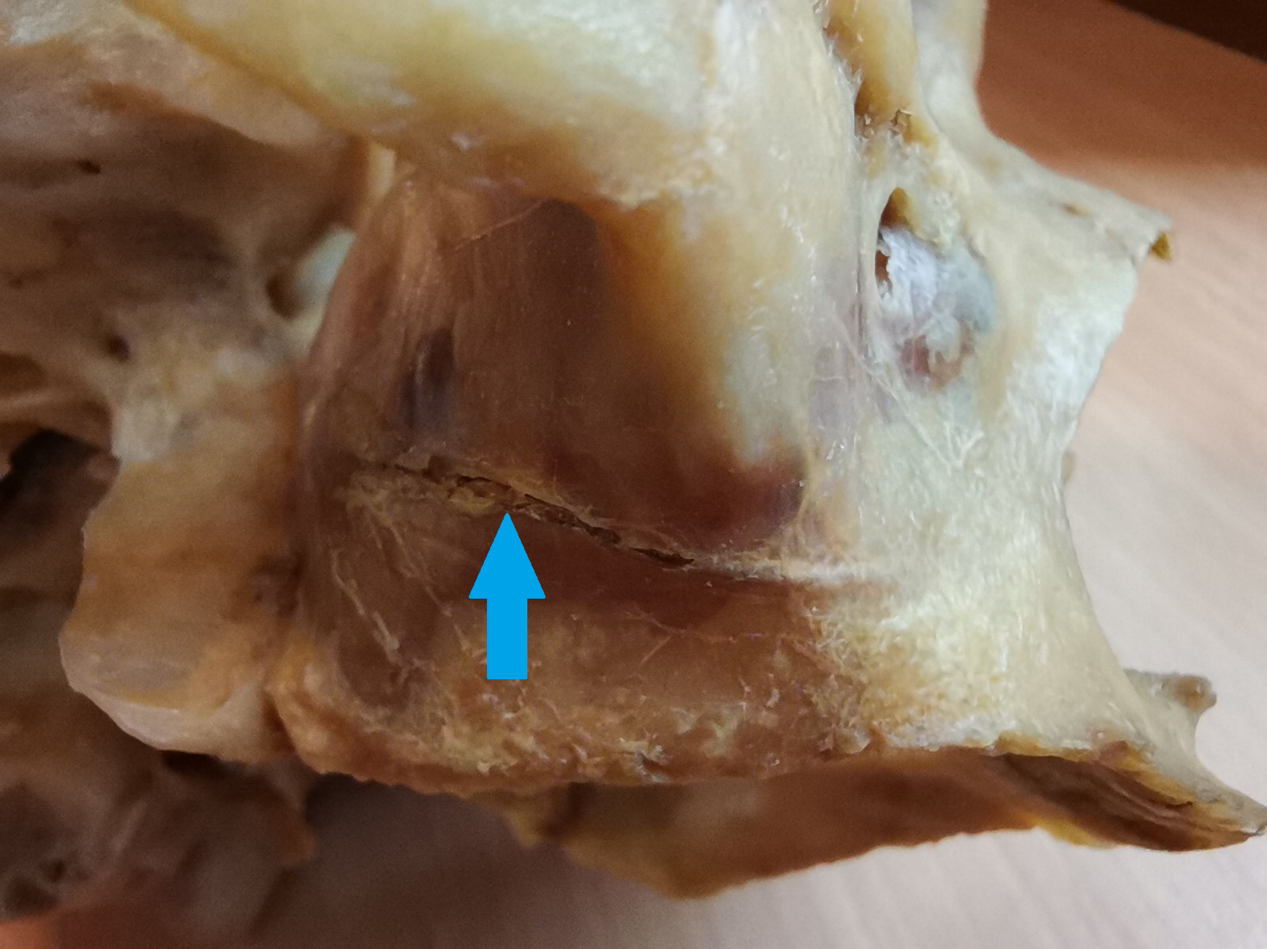
The alveolar antral artery enters the bony canal on the skull. The bony canal is marked by a blue arrow.

Lateral wall osteotomies can interfere with AAA approximately in 20% (Elian et al., 2005). Figure 1 shows the collision of the lateral sinus lift window and the AAA on the skull from the anatomical institute. This window was artificially created to illustrate the dangers of this surgical procedure. Figures 2 and 3 show the course of the bony canal in which the AAA runs on the skull and its position relative to other anatomical structures.

**Figure 4:**
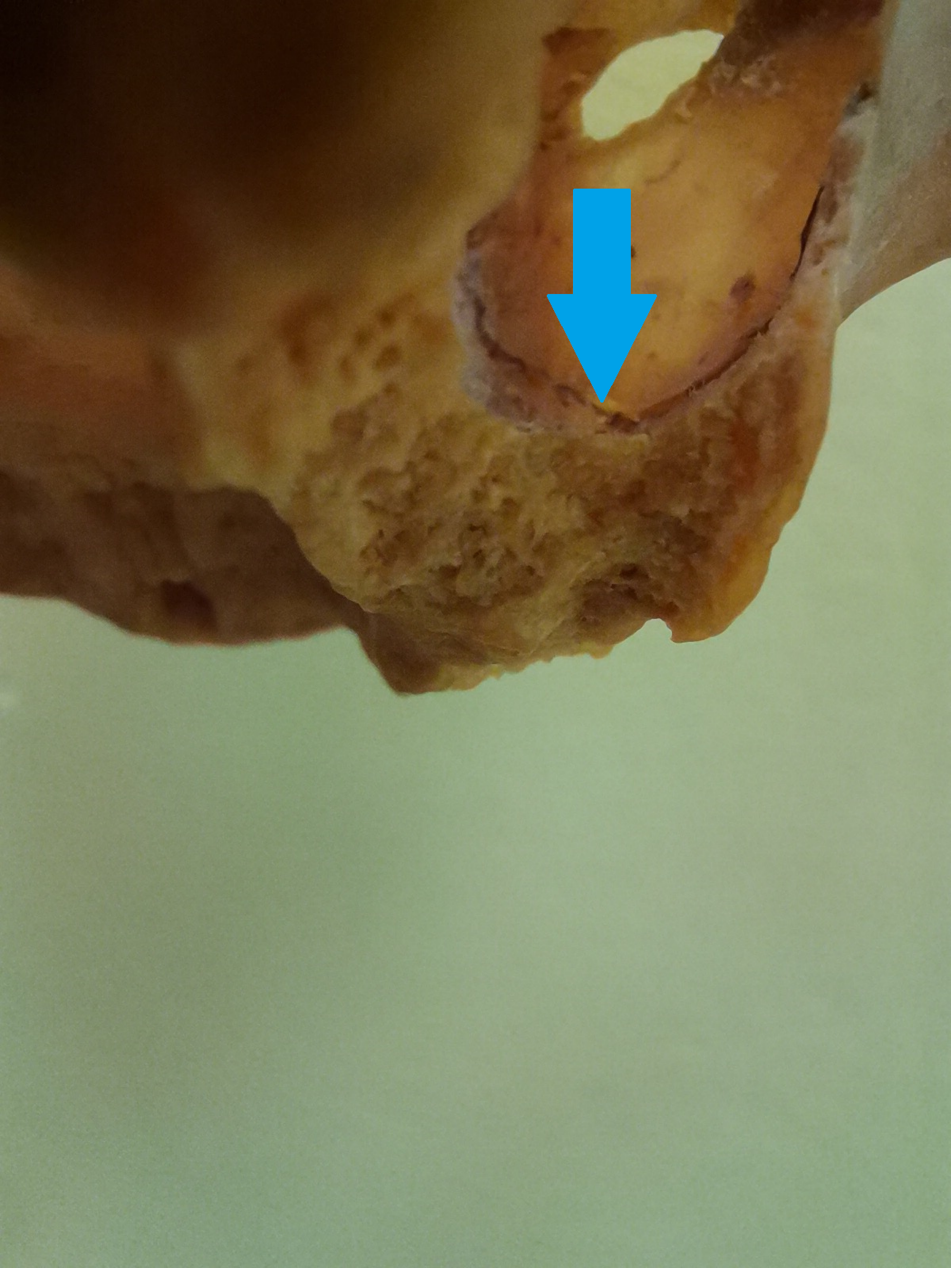
The view into the maxillary sinus of the skull from the posterior side, the alveolar antral artery in the intrasinus form is visible.

**Figure 5:**
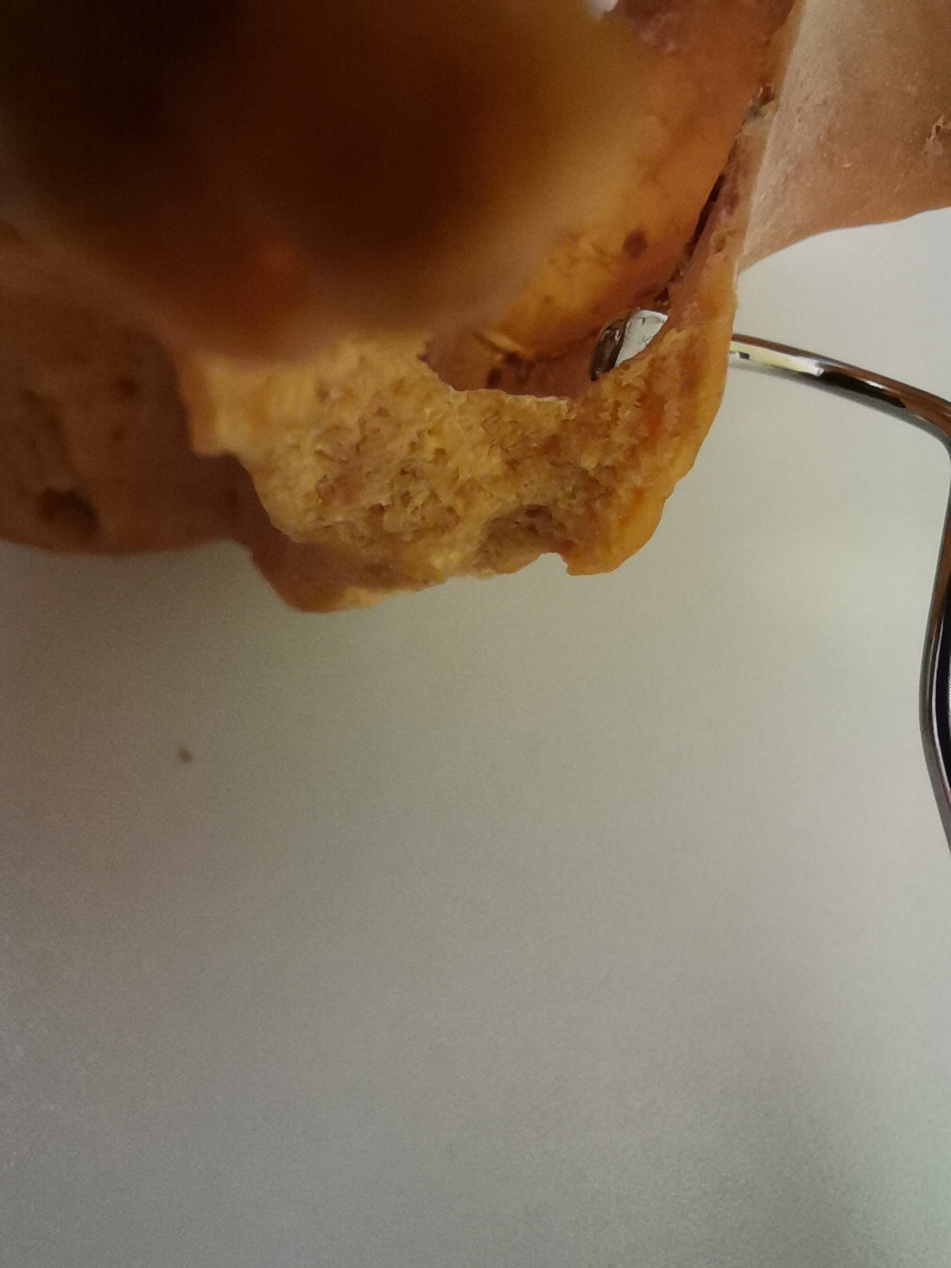
Sinus lift curette in the same sinus to demonstrate its work and danger.

**Figure 6:**
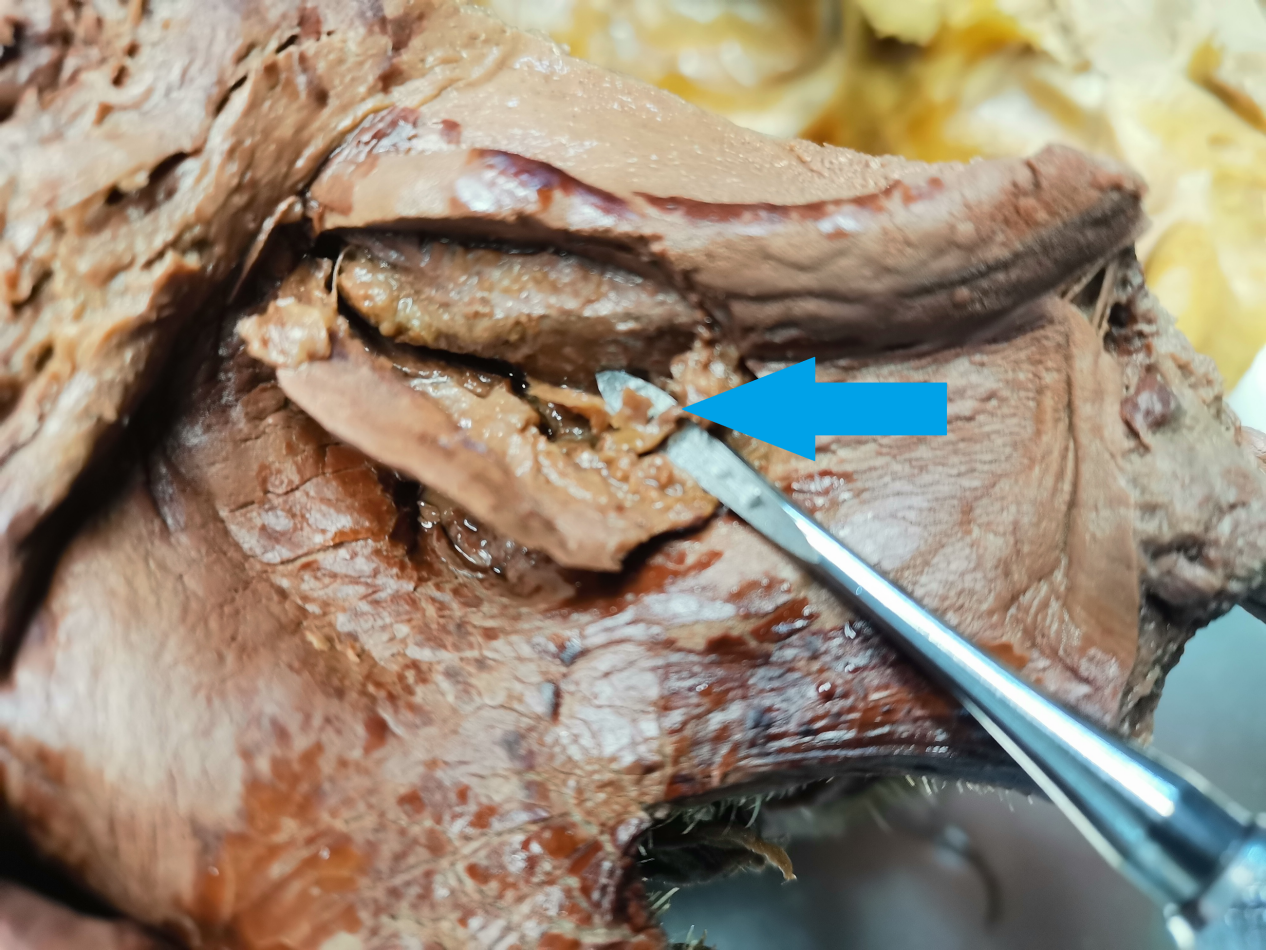
Alveolar antral artery during autopsy. The AAA is marked by a blue arrow.

The perioperative hemorrhage could be immediate or delayed (hours) after surgery due to vasoconstrictors (Balaguer-Martí et al., 2015). Diameters of AAA to 0, 5 mm does not represent a severe hemorrhagic risk (Rysz et al., 2014). Not always the clinical findings correspond to the radiographic picture, in in small radiological finding, the large diameter vessel could be found (Testori et al., 2010). There are three possible variants: extrabony AAA, intrabony and intrasinus. Only intrabony form can be detected on CBCT. That can be explanation of discrepancy between cadaver and CBCT studies (Rysz et al., 2014). Figures 4 and 5 show AAA during its course in the sinus maxillaris. In Fig. 5, the curette is located below the vessel, demonstrating the sparing of the vessel during surgery. Figure 6 shows the extrabony form AAA on the cadaver.

**Figure 7:**
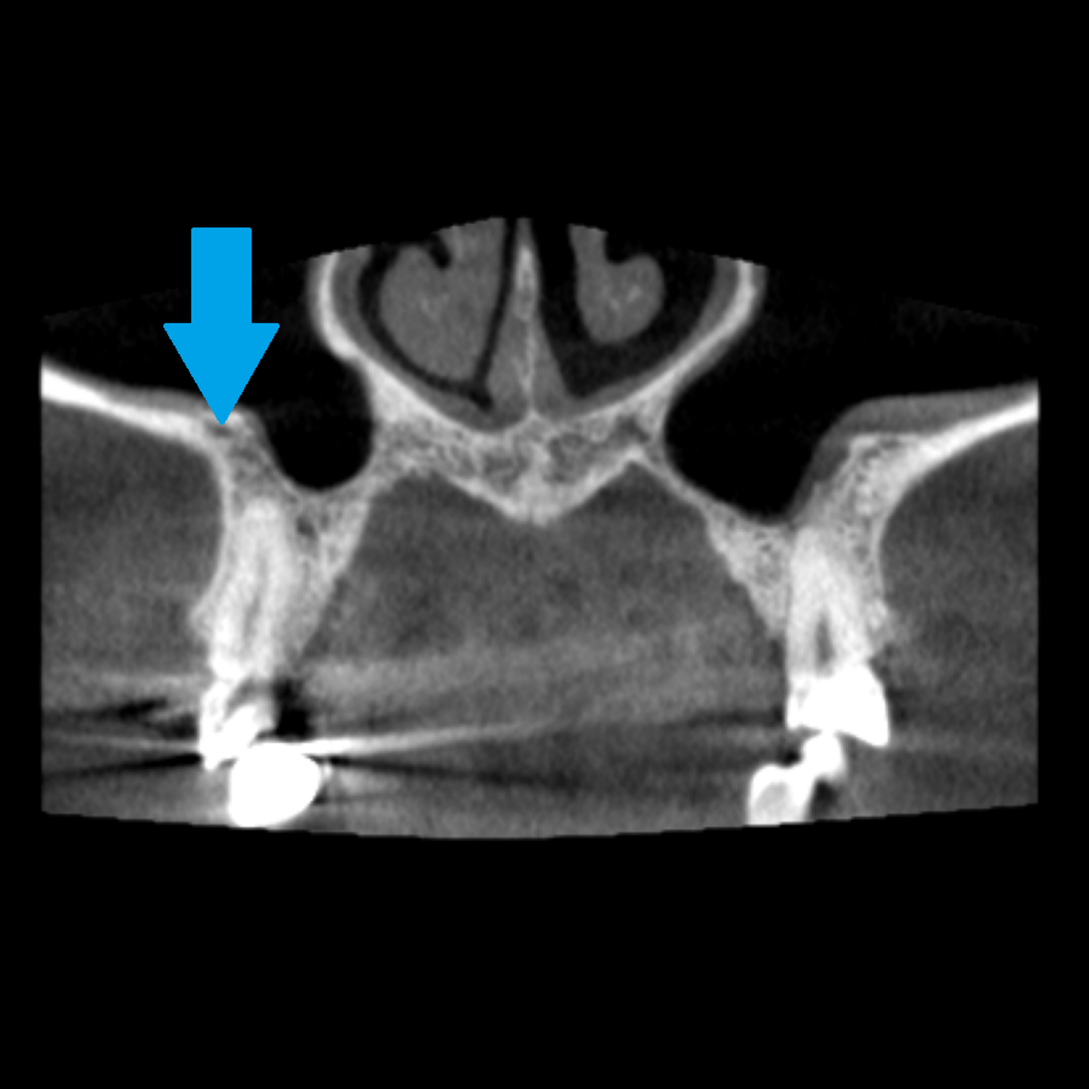
Alveolar antral artery on the CBCT, coronal view. The blue arrow marks the position of bony canal containing AAA. The bony canal is more visible in dynamic viewing, in static images it can be confused with the cavity in the spongiosis.

The intrasinus alveolar antral artery presents a difficulty in bleeding management. The bleeding during window preparation can jeopardize the visibility and the surgery is more prone to mistakes. There is a risk of severe hemorrhage in the presence of larger canals (larger than 3 mm) (Rahpeyma & Khajehahmadi, 2014). Figures 7–Fig. 9 demonstrate AAA bone canal of different dimensions on CBCT.

**Figure 8:**
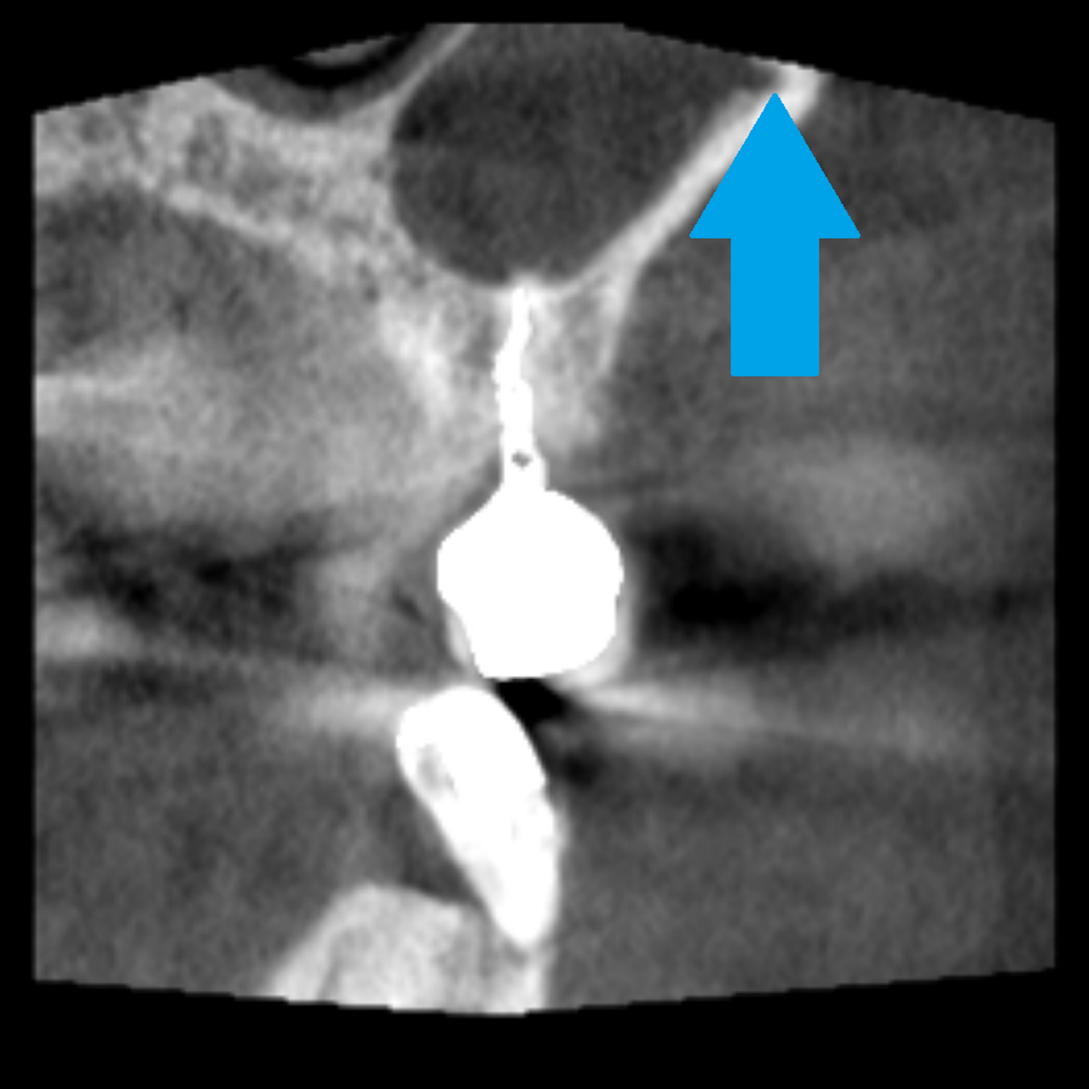
Coronal view of the CBCT, large diameter of the alveolar antral artery coronally from dental implant. The blue arrow marks the position of bony canal containing AAA.

**Figure 9:**
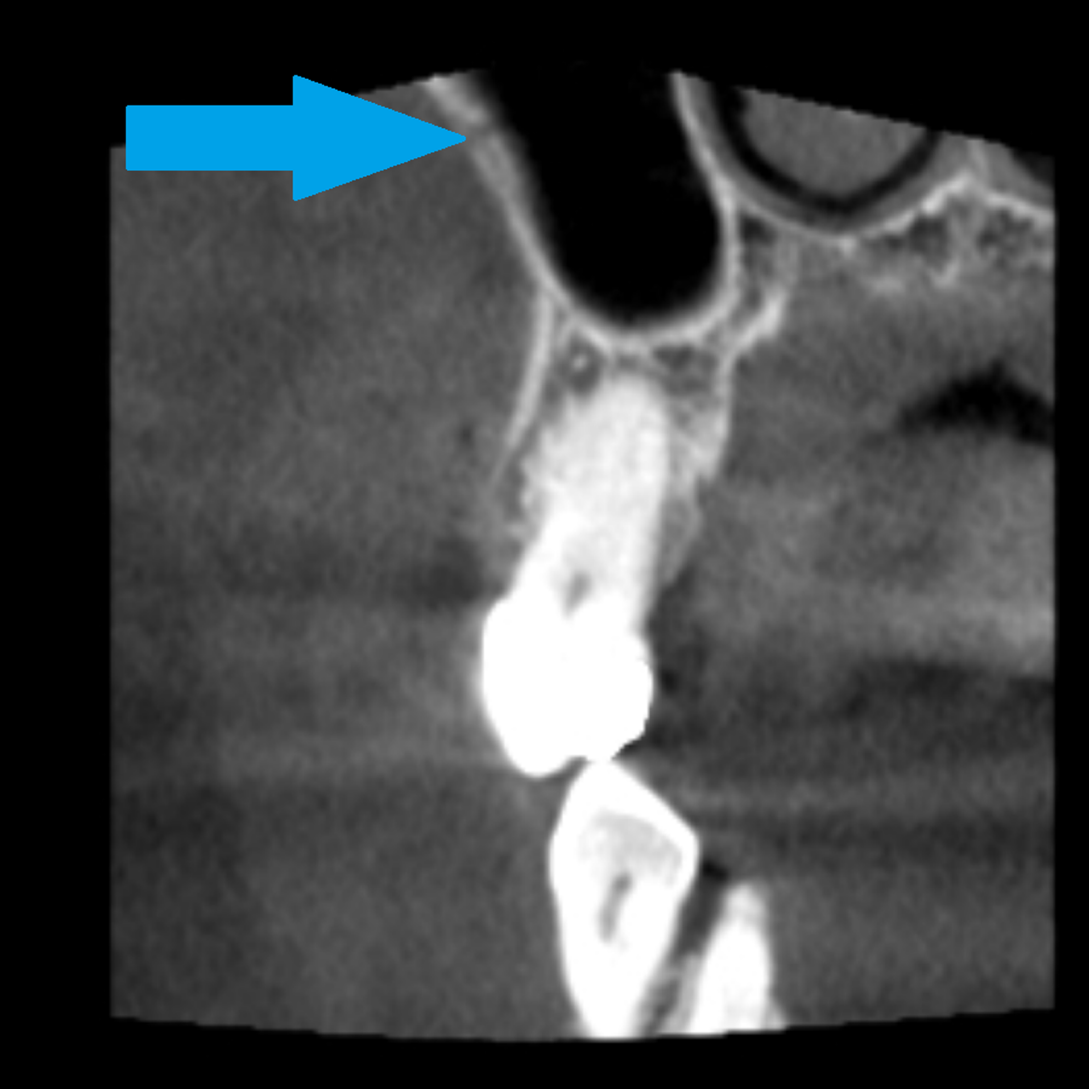
Bony canal of alveolar antral artery on the CBCT, coronal view, AAA is marked by a blue arrow.

Although the nerves can accompany the AAA in bony canal, after trauma of AAA, there is no risk for involving maxillary teeth sensitivity (Murakami et al., 1994). Preservation of the AAA can hypothetically important for blood supply of the periosteum of maxillary sinus and for neo-angiogenesis to the bone graft from periosteum. This is discussed in the discussion (Taschieri & Rosano, 2010). Adequate prevention of the arterial bleeding or alternative surgery can be performed.

Appropriate management of the arterial bleeding should be followed. This is an anatomical study describing characteristics of alveolar antral artery and its relation to neighboring structures. In discussion part clinical management of challenging situations will be described.

## Materials & Methods

### Design

This is an observation study.

### Settings

The study protocol was reviewed and approved by the Ethics Committee of the Faculty of Medicine and Dentistry, Palacky University Olomouc Ref. No. 81/22. The informed consent of using radiographs and photos of the patients for scientifical and research purposes is routinely signed in the Institute of Dentistry and Oral Science Palacky University and was provided to the journal Editorial office.

Exclusion criteria:

1. Invalid CBCT scan or CBCT scans which does not include any maxillary sinus.

2. The CBCT scans imaging only one maxillary sinus was included, but excluded from analysis of bilateral presence of intrabony AAA. If one person had multiple CBCT scans the more aimed for sinus maxillaries was chosen.

3. Presence of maxillary sinus pathology such as cyst or pseudocyst which does not allow further measurement.

4. Grafted alveolar sinus.

The 251 CBCT scan was evaluated. 103 CBCT scans was excluded for inadequate imaging (60), artefacts (40), pathology (three), if the sinus was grafted, the preoperative CBCT scans were found and used.

The 148 valid CBCT scans of patients from university hospital databases was examined. The CBCT scans was done for different reasons such as implant therapy planning or advanced diagnostics.

**Figure 10:**
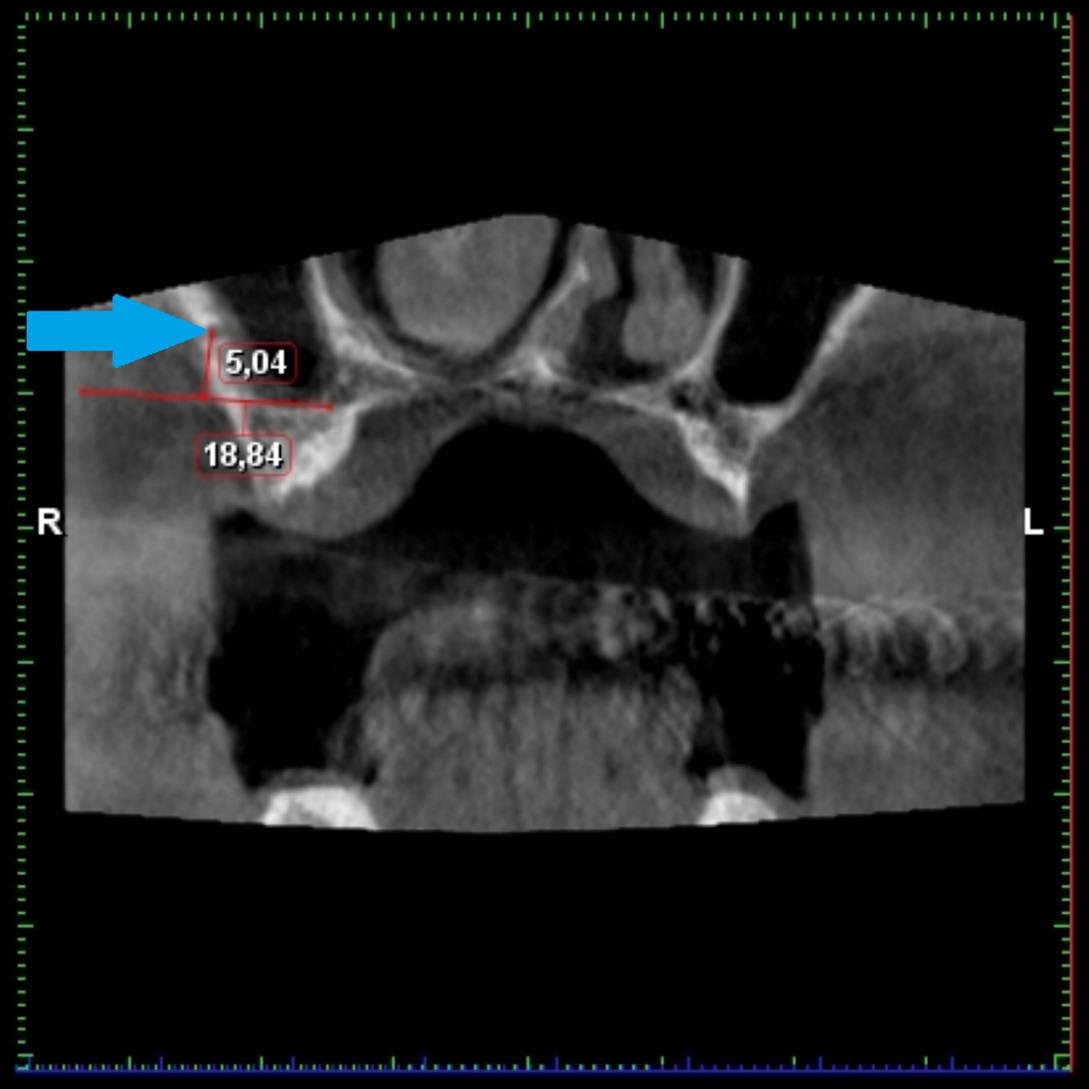
Demonstration of the measuring of the distance from the sinus floor. AAA is marked by a blue arrow.

The data of the patients was obtained (gender, age). The quality of CBCT scans was verified. The possibility of bilateral examination of maxillary sinus was ensured. If not, this was noticed. Presence and bilateral presence were examined. The distance between AAA and sinus floor was measured in mm in first premolar (P1), second premolar (P2), first molar (M1) and second molar (M2) region was measured. Figure 10 illustrates the methodology for measuring the distance between the sinus maxillaris and the AAA.

The high and width of bony canal was measured. Width of sinus wall in M1 region was measured to study the relation of sinus wall width and bony canal diameter.

The distance of AAA from the level of hard palate was measured in M1, P2 and P1. (Notice no M2). Missing teeth were noticed.

**Figure 11:**
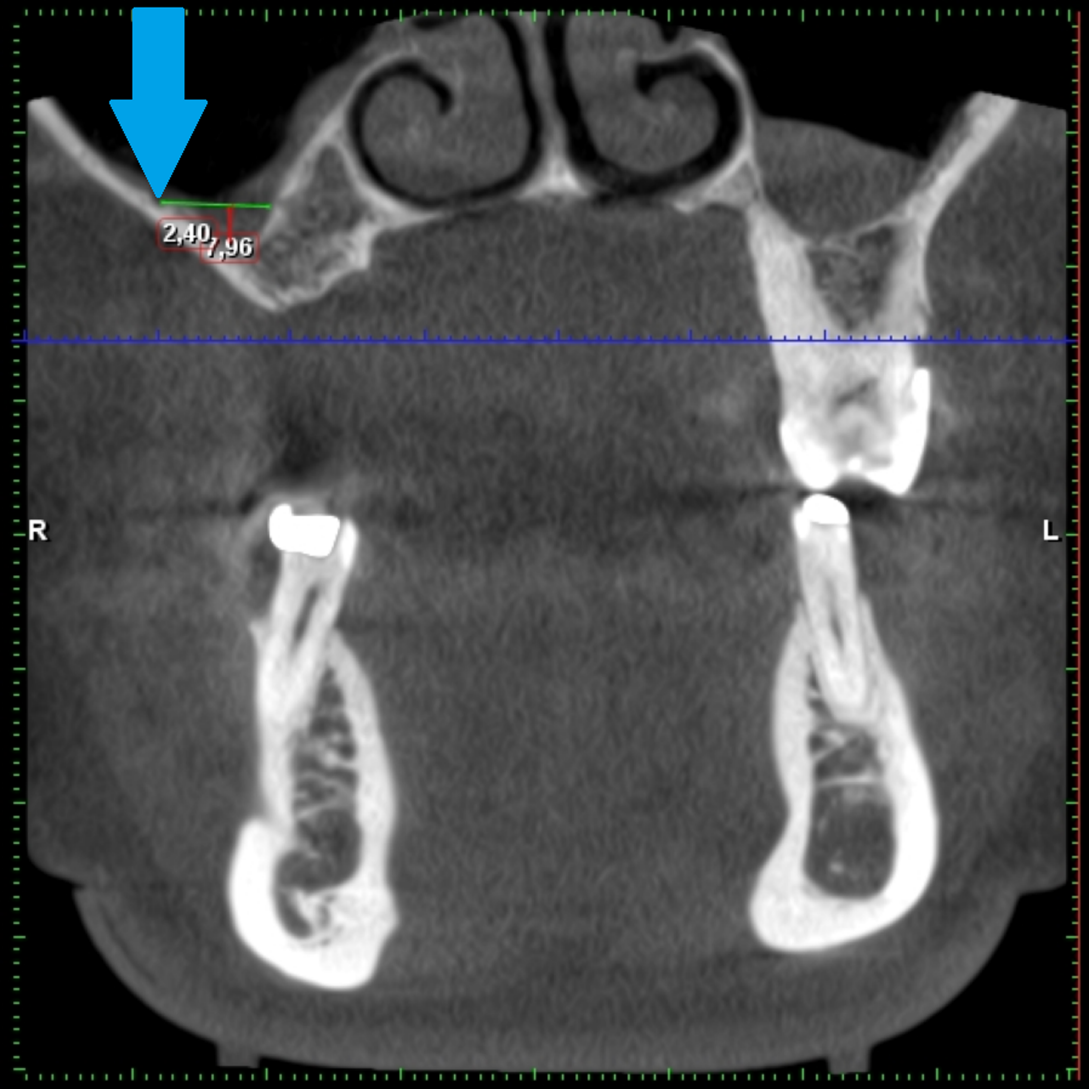
Demonstration of the kind of the sinus measuring according to Chan. AAA is marked by a blue arrow.

The sinus type (wide, average, and narrow) was measured. Classification of sinus in width was based on Chan et al. (2014). The sinus width at lower boundary (2.3 mm from sinus floor) was measured. If the sinus width was smaller than 8 mm, the sinus was classified as narrow, the diameter from 8 to 10 mm was classified as average. A sinus wider than 10 mm was classified as wide (Chan et al., 2014; Whyte & Boeddinghaus, 2019). Figure 11 shows the measurement of the cavity type according to Chan et al. (2014).

**Figure 12:**
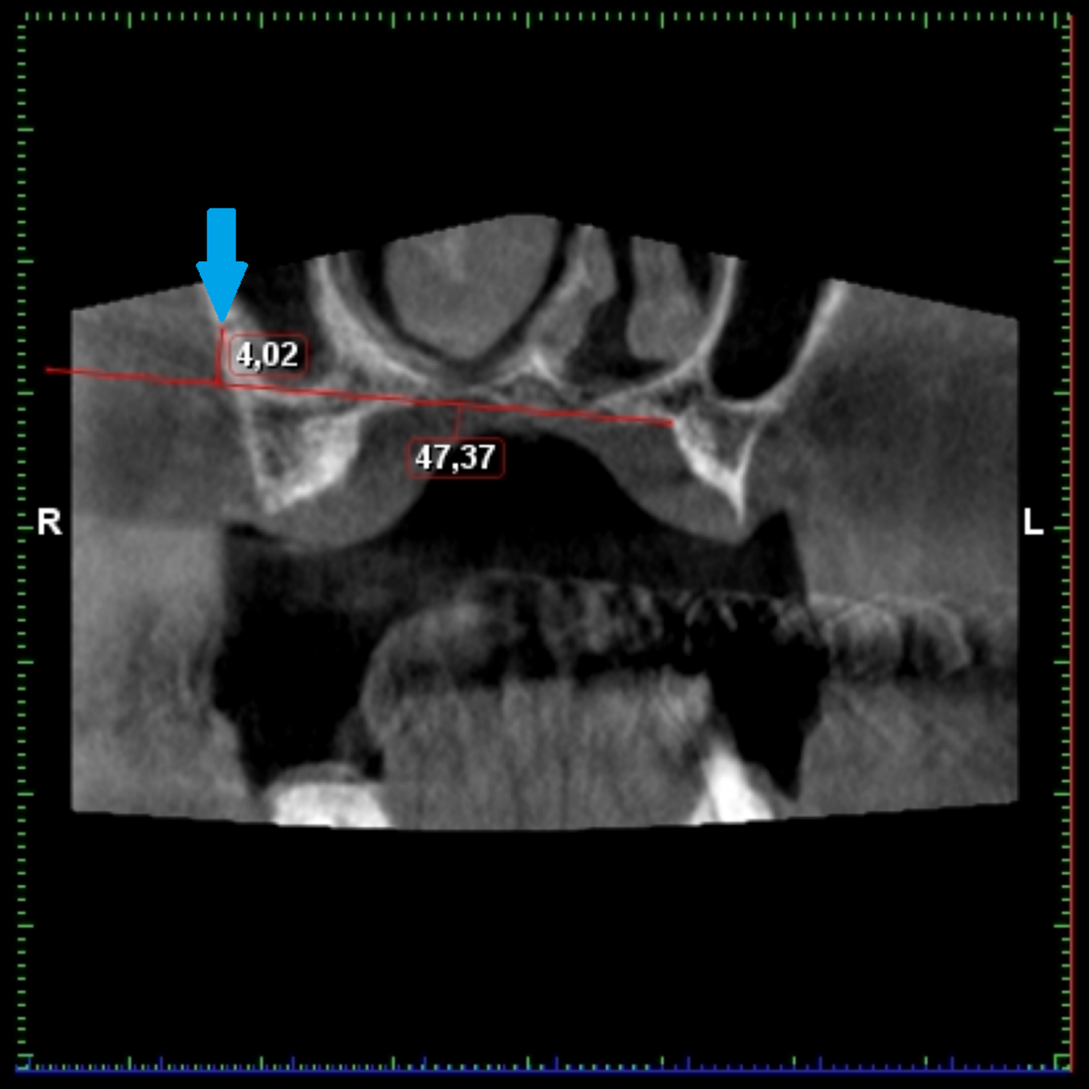
Demonstration if the measuring of the distance from the hard palate level. AAA is marked by a blue arrow.

Bifid and trifid branches of AAA were found in more cases. However, no statistics were kept on the incidence of bifid or trifid AAAs and the most caudal branch, closest to the alveolar process and thus with the greatest significance for sinus lift, was always used for measurement. Figure 12 demonstrates the measurement of the distance of AAA from the plane of the hard palate.

## Results

Alveolar antral artery was found in 69 cases (57.0%); in the group of man it was in 27 cases (65.9%) and in the group of women in 42 cases (52.5%). The hypothesis of age dependance of bony canal presence was tested. The patients were divided into three groups of different age 1. younger than 20 years, 2. between 21–40 years of age and 3 older than 40 years. The percentual increase in older patients was observed. However statistical significance was not found. The presence of bony canal in group 1 was 50%, in group 2 was 58.8% and in group 3 was 63.6%. This data are visible in Table 1.

**Table 1:** Presence of bony canal in lateral maxilla and its relation to age. Data shows the raise of presence of the bony canal in maxillary wall in higher age. This was not statistically significant

	Presence of bony canal	No bony canal observed	*p*-value
1. Up to 20 years	29 (50.0%)	29 (50.0%)	0.379
2. between 21–40 years	10 (58.8%)	7 (41.2%)	
3. Older than 40 years	28 (63.6%)	16 (36.4%)	

Although the more AAA in bony canal was found in younger patients, the percentual occurrence was higher in the older group. This was caused by the number of young patients. This is visible in Table 2.

**Table 2:** Age of the patient with bony canal presence and without its presence. Data shows median age of the patients with and without bony canal. The relation of the age to the occurence of the bony canal was not statistically significant. Median is influenced by group of patient with CBCT from orthodontic reasons, typically younger patients.

Age	Average	Median	Min − max	Mann-Whitney test *p*-value
AAA in bony canal	29.3	18.0	8–81	0.064
No bony canal detected	36.1	31.0	9–91	

The hypothesis of the presence of bony canal and type of the sinus was tested. The three types of sinuses are narrow, average, and wide. Null hypothesis can be statistically confirmed. However the wider the sinus is, the more likely the AAA will be found. The numbers of patients with different types of sinus maxillaris and the presence of AAA can be seen in the Table 3.

**Table 3:** Type of maxillary sinus and bony canal presence. There is no correlation between sinus width and radiographic presence of artery.

	Presence of bony canal	No bony canal detected	Test *p*-value
N −narrow	14 (93.3%)	1 (6.7%)	0.734
A − average	19 (95.0%)	1 (5.0%)	
W − wide	19 (100%)	0 (0.0%)	

The average distance from hard palate level in the region of first molar, first premolar and second premolar was measured. In the M1 region the AAA in average goes 2.6 mm above hard palate level. And it goes cranially to P2 a and P1 region. The increasing variability can be seen in Table 4.

**Table 4:** Distance of alveolar antral artery from hard palate level. Average, standard deviation, median and minimal and maximal values are shown. M1 region is relatively stable, P1 region shows the biggest variability.

	Aaverage	S	Median	Min − max
Distance of the AAA in M1 region	2.6	3.4	2.4	−3.2 to 16.8
Distance of the AAA in P2 region	3.2	3.8	2.4	−2.5 to 17.7
Distance of the AAA in P1 region	7.7	4.7	7.1	0 –21.6

For data analysis the software IBM SPSS Statistics version 23 (IBM Corp., Armonk, NY, USA) was used. To assess the dependence between the occurrence of the AAA and age (age intervals), or between the occurrence of the vessel and the type of the sinus, the chi-square test was used, or Fisher’s exact test. The Mann–Whitney U test was used to compare age in the group with and without vessel occurrence. Data normality was assessed using the Shapiro–Wilk test. All tests were done at the 0.05 significance level.

The probability of bilateral presence of bony canal was tested. Not every CBCT scan allowed biliteral reading and maxillary sinus. CBCT in which AAA could be detected showed bilateral osseous canal in 52.8%. Details can be found in Tables 5A and 5B In the M1 region, no more than 1 mm difference was found between the left and right AAA in relation to the plane of the palatal plate. In regions P1 and P2, this dimension was not evaluated or further statistically processed.

**Table 5:** (A) Bilateral imaging and bilateral presence of AAA; (B) unilateral imaging and unilateral presence of AAA. Bilateral presence is not the rule. Our study found bilateral presence if the AAA in 60%.

(A) Bilateral imaging and bilateral presence of AAA		
			No	
	Amount	Percentage	Amount	Percentage
The CBCT shows both sinus maxilaris?	72	60.5%	47	39.5%
Is AAA present bilaterally	34	47.2%	38	52.8%

(B) Unilateral imaging and unilateral presence of AAA		
	Amount	Percentage
Bilateral imaging and presence of AAA	31	43.1%
Unilateral imaging and presence AAA	20	27.8%
Unilateral imaging and no presence AAA	21	29.2%

The distance between the floor of the maxillary sinus and the AAA in the area of P1, P2, M1 and M2 was measured. Average distance in the M1 region was 8.4 mm in P2 7.3 mm. As those are clinically the most sinus lift demanding areas. For detailed results, see Table 6.

**Table 6:** Distance of the AAA and the sinus floor, average, standard deviation, median, minimum and maximum.

	Average	SD	Median	Minimum	Maximum
Distance of the AAA from sinus floor in the area of P1	6.4	3.3	5.5	0	15.3
P2	7.3	3.9	6.0	0	18.2
M1	8.4	4.6	8.2	0	17.1
M2	8.8	4.7	9.8	0	21.0

The average width and height of bony canal was measured. The relationship of the diameter of the canal and age and gender were measured. Tables 7A–7D. Average height and width of the bony canal was found 0.9 mm. There was found no correlation between canal width and age. Canal width was found significantly bigger in group of man (*p* = 0.015). The width of the sinus wall from the bottom of the sinus in the area of M1 was measured.

**Table 7:** Characteristics of the AAA based on maxillary wall thickness, age and patients gender.

(A) Height, width of AAA and the thickness of sinus wall in M1 region.					
	Average	SD	Median	Minimum	Maximum
Height of bony canal	0.9	0.3	0.9	0.5	1.7
width	0.9	0.3	0.8	0.5	1.8
Width of sinus wall in M1 region	2.4	1.8	1.7	0.6	8.9

(B) The diameter of the AAA and age	
Age *vs* width of the canal	
Correlation Coefficient	0.021
p	0.872

(C) The diameter of the AAA and gender				
Width of the canal	Median	Minimum	Maximum	*p*
man	0.9	0.5	1.8	0.015
woman	0.8	0.5	1.4	

(D) The diameter of the AAA and the width of sinus wall in M1 region	
Width of sinus wall in the M1 vs.	Canal width
Correlation Coefficient	−0.046
*p*	0.742

No significant relationship between canal width and sinus width was demonstrated. This is a result which differs from other studies (Rahpeyma, Khajehahmadi & Amini, 2014). No significant relationship between sinus type and AAA in the M1 region was demonstrated in the group of patients with missing teeth or in the group without missing teeth. Although the group is small for statistical purposes; we can say we cannot prove the sinus change in width after tooth loss. The distance of the AAA from the mandibular floor was studied in patients with teeth still present and in patients who had lost teeth, in patients with different types of maxillary sinuses. The results are shown in Tables 8A and 8B. These results are not statistically significant but are very close to the significance level.

**Table 8:** The distance of the AAA and sinus floor and its relation to tooth loss. The distance of the AAA and sinus floor increases after tooth loss as the sinus is pneumatizing the alveolar ridge.

(A) The distance between AAA and the sinus floor in edentulous patients.			
Distance of the AAA an the sinus floor in the region	Median	min - max	*p*-value
Sinus A (*n* = 8)	7.3	6.6–12.0	0.793
Sinus N (*n* = 8)	12.9	4.0–14.0	
Sinus W (*n* = 9)	10.4	6.1–14.5	

(B) The distance of the sinus floor and AAA in patients with teeth present.			
Distance of the AAA and sinus floor in M1 region	Median	min − max	*p*−value
Sinus A (*n* = 11)	4.9	0.0–11.7	0.067
Sinus N (*n* = 7)	13.0	1.6–14.3	
Sinus W (*n* = 9)	7.0	1.0–8.9	

## Discussion

### Study design

During the design of the study authors decided use retrospective data and not to use diagnostic methods which are not indicated from another medical reason. This is cost consuming and patient should be avoided of unnecessary ct scanning. Authors decided not to use MRI for study for high presence of artefacts caused by dental materials.

**Figure 13:**
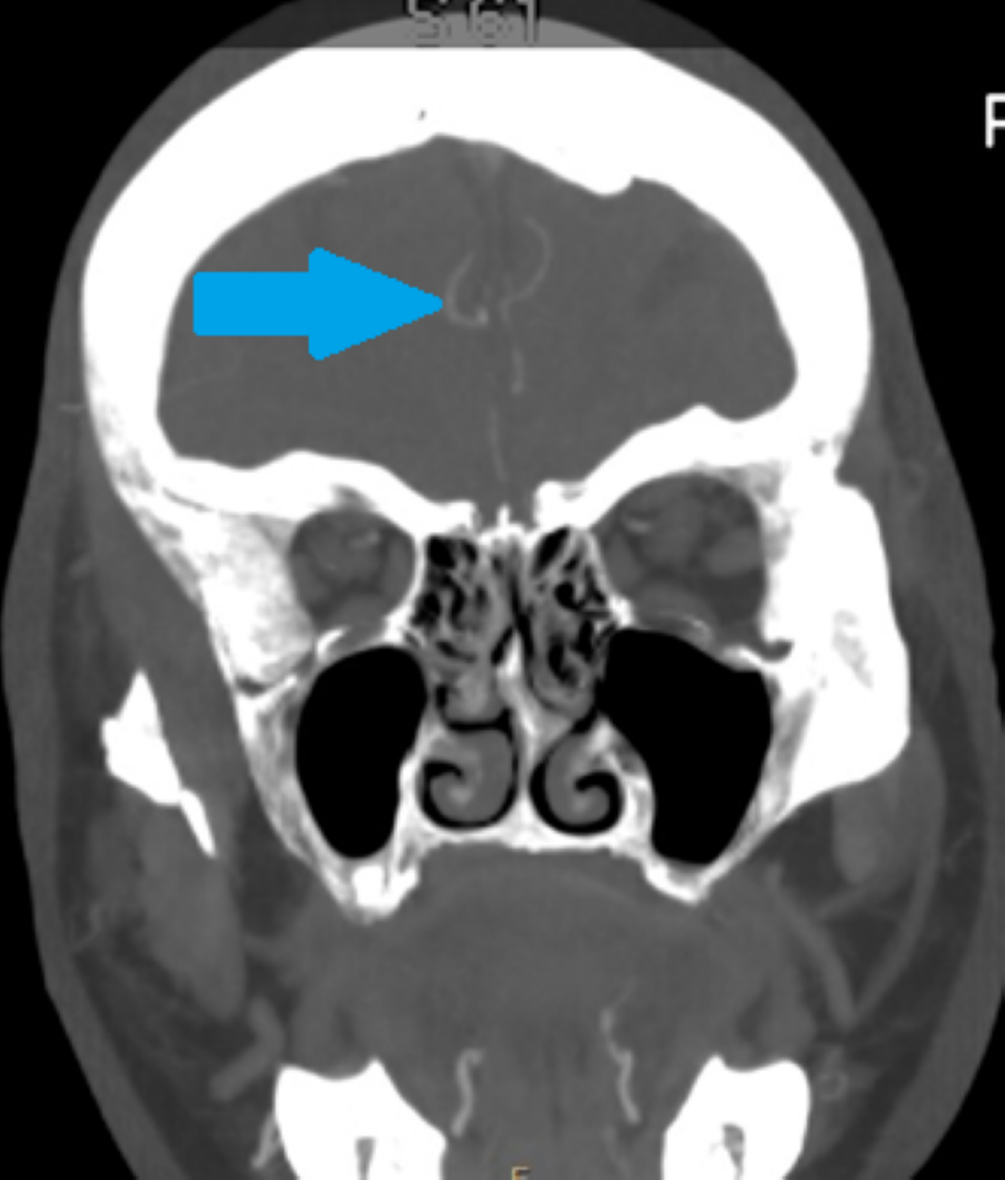
CT angiography, coronal plane. CT angiography, this diagnostic method is aimed for the large diameter brain vessels (marked with a blue arrow) and unfortunately not suitable for AAA detection.

As possibility the authors considered Ct angiography, which is indicated for stroke diagnosis. However, the contrast material applied intra venosus does not shows AAA as the diagnostic method is indicated for brain vessels with higher diameter. Figure 13 demonstrates the low sensitivity of CT angiography in the splanchnocranium.

As dental clinicians use CBCT for routine diagnostics before implant placement, the authors use the same diagnostic method to evaluate presence and characteristics of AAA.

### Results

In our study, 0.9 mm was found as average diameter of the AAA. This correlate with other studies. For example, Güncü et al. (2011) found mean diameter of AAA was as 1.3 ± 0.5 mm.

In our study, 57% of the measured CBCT scans contained AAA. We found the bilateral presence in 43%. This is needed in order to compare with other teams work where AAA was present in 88.5% of the assessed maxillary sinuses and bilateral visualization was predominant 77% (*P* < 0.0001). The mean vertical distance from the most anterior part of the AAA to the sinus floor was 7.9 ± 6 mm in female patients (both sides) and 12 ± 7.22 mm on the right side and 10.9 ± 6.86 mm on the left side in males, where they had maximum. The mean diameter of the AAA was 1.2 ± 0.7 mm on both sides in females. In males, the diameter was significantly (*P* < 0.05) larger: 1.5 ± 0.62 mm on the right side and 1.4 ± 0.69 on the left side in females (Albuquerque et al., 2021). This agrees with our study, as also confirmed by the higher width of the AAA in male group.

In our study was found higher distance between sinus floor and AAA in edentulous patients *p* = 0.793 than patients with teeth present *p* = 0.067. However, the same distance (in both edentulous or non-edentulous patients) was found at the hard palate level. Hypothesis can be done that dental roots or periodontal ligaments are protective for the alveolar ridge and after the tooth loss, the alveolar ridge becomes pneumatized. The significance of this finding contributes to a better understanding of maxillary sinus development. Clinically, after tooth loss (but not necessarily with respect to anatomical variability), maxillary sinus surgery may be more compromised by AAA bleeding than maxillary sinus surgery in a patient with teeth still present.

Presence of the bony canal in the lateral wall of maxillae showed statistical probability depending on age with *p* = 0.064. This is not statistically significant, but it is very close to the border *p* = 0.05. In our study was shown strong correlation for older patients to have AAA present in lateral wall of maxilla.

### Practical management

The AAA presents danger for operator and for the patient; short time result can be jeopardized by severe bleeding and decreasing visibility, which can result in perforations and mistakes (Taschieri & Rosano, 2010).

First of all, preservation and minimal invasivity is the key approach in dentistry. We know the AAA is 100% present and in 20% it can be in the way of lateral sinus lift. To avoid injury, different techniques can be considered: short implants or transcrestal sinus lift. However, these techniques also have their limitations. When making decisions, it is also important to consider the long-term prognosis of the treatment and not to harm the patient with an inferior treatment outcome (Ella et al., 2008; Sedov et al., 2020).

If the lateral sinus lift is necessary or highly advantageous and AAA is in the way of surgery, careful planning and osteotomy shifting, is advantageous (Cecchetti et al., 2021).

If there is no possibility to shift the osteotomy, the kind of the AAA should be considered. In case of intrasinus AAA, the piezo surgery is possible and avoiding the damage of the artery is possible. In the case of intrabony canal, the double window technique is advantageous. The smaller lateral window osteotomy can be advantageous. Extrabony AAA is raised with flap, the avoiding of damage is important during releasing the flap. The use of scissors is possible (Maridati et al., 2014).

**Figure 14:**
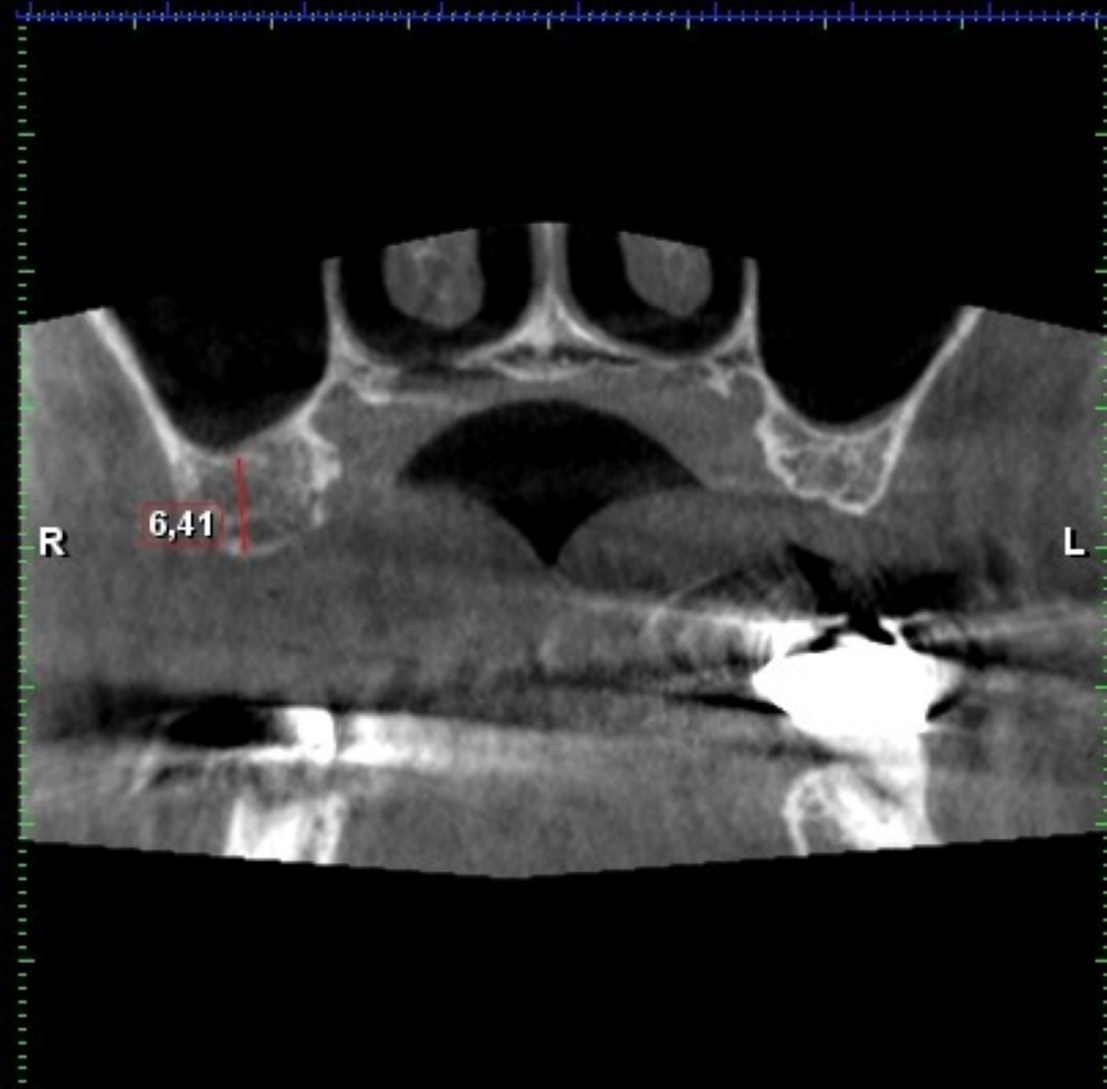
Residual bone high in the first molar region (6, 4 mm). AAA is not present on this CBCT.

**Figure 15:**
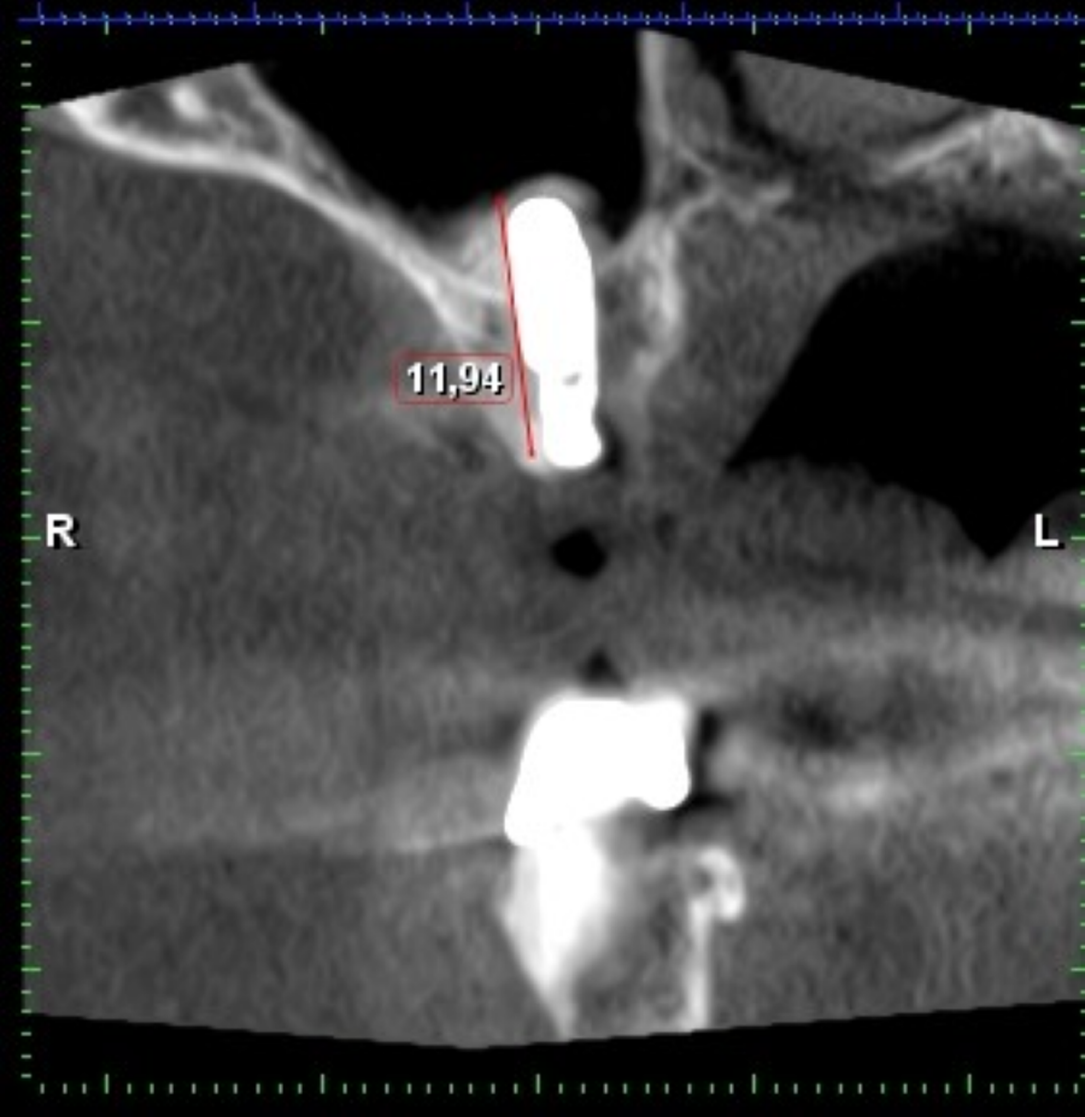
CBCT after internal sinus lift with implant placement. Internal sinus lift using A-prf, sticky bone and versah kit allowed 12 mm long fixture without perforation. The bony canal containing AAA is not clearly visible on this CBCT. This CBCT is illustrative and because of grafted sinus was excluded from research.

In case of severe bleeding from artery in diameter larger than 3 mm, the preventive ligature can be considered or flap elevation with preparation to ligature of distal aspect, can be considered (Yang & Lee, 2021). Figures 14 and 15 show the result of an alternative surgical approach—an internal sinus lift which is also a very conservative treatment option, albeit with some limitations—was used to elevate the floor of the maxillary sinus by six mm.

If the diameter is smaller than 0.5 mm, no management is possible option (Yang & Lee, 2021).

In case of relatively severe bleeding, the kind of the AAA varies in management. In extrabony component, the electrocautery or ligature is possible. In the case of intrabony, the compression or obstruction of the orifice is possible. The intrabony component can be combined with extrabony or intrasinus, the obstruction may not be effective in incomplete bony canal. Intrasinus is challenging: the electrocautery can lead to damage of Schneiderian membrane, and ligature may not be possible. Finding the intrabony component and its management can be an effective course of action (Maridati et al., 2014; Yang & Lee, 2021).

**Figure 16:**
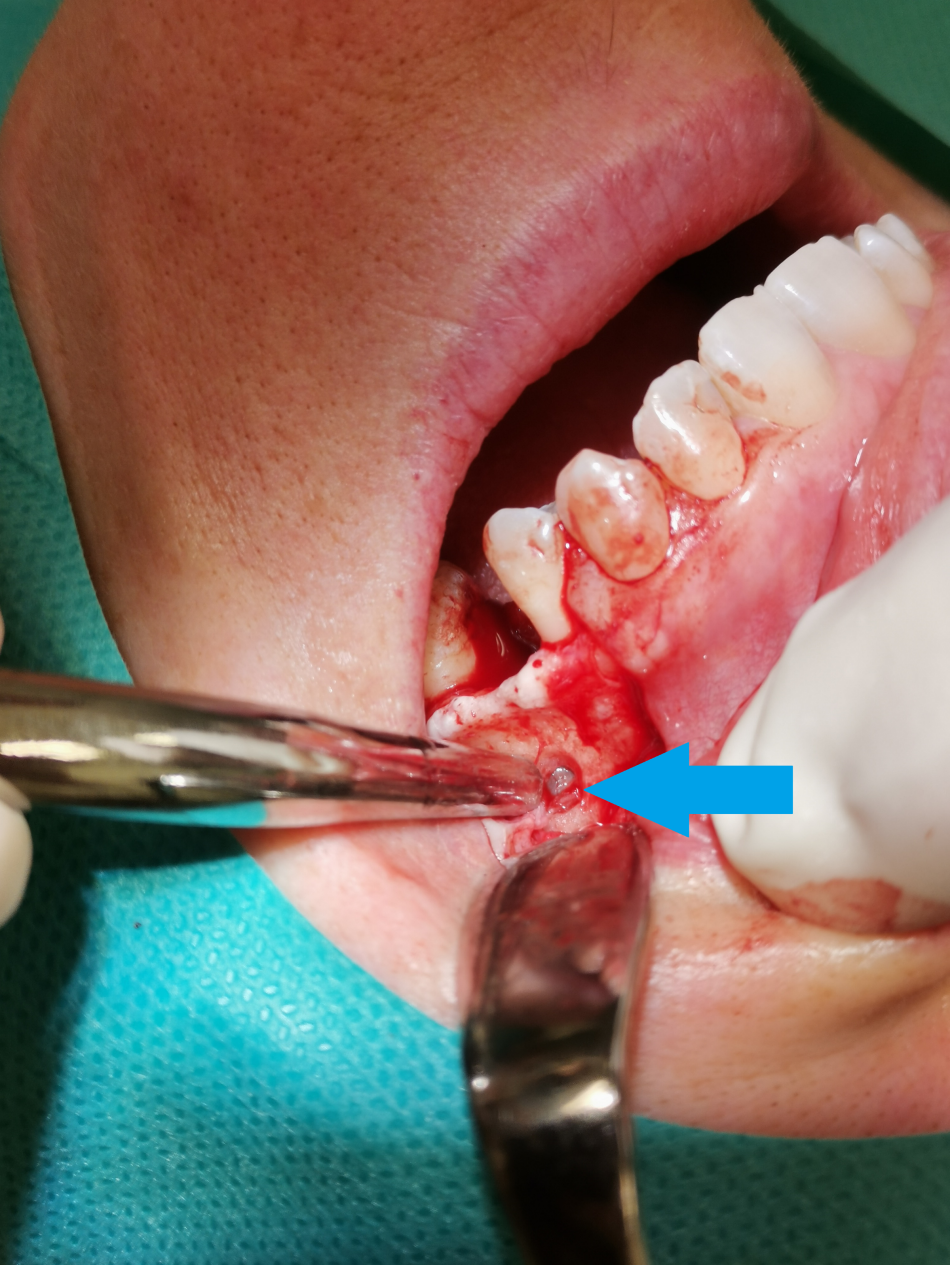
The alveolar antral artery in the intrasinus form during operation. The alveolar antral artery in the intrasinus form during operation. The AAA is marked by a blue arrow.

Figure 16 demonstrates an intact AAA in the lateral sinus lift window osteotomy. Comparison of maxillary sinus floor distances in edentulous and edentulous patients provides insight into maxillary pneumatization, which appears to be inhibited by the presence of tooth roots. Thus, in edentulous patients, the distance of the maxillary sinus floor from the at-risk AAA structure increases. This should be considered, for example, in orthognathic surgery where the bone incision is guided over the teeth. The relationship between the position of the hard palate, which is not subject to resorptions, and AAA has not shown a significantly stable relationship, especially in the P2 and P1 positions, where AAA occurs with considerable variability, thus indicating the need for a thorough examination. However, proximity of the AAA to the alveolar process rather than mesially is common in the M1 region.

There is no direct evidence that limiting maxillary periosteal nutrition by sacrificing AAA could compromise the success of bone grafting. This hypothesis was put forward by Taschieri & Rosano (2010). However, the maxilla is a highly vascularized bone. The very thin maxilla in the lateral section obviously lacks vascular supply from the spongiosa, but it is also nourished by the periosteum on the other side (Taschieri & Rosano, 2010).

## Conclusions

The knowledge of the risks of arterial bleeding during sinus lift surgery and the minimally invasive decision making can be beneficial for patient and clinician. The characteristics and description of alveolar antral artery can increase the surgeon knowledge and led to more careful approach.

After tooth loss, the maxillary sinus expands into maxillary alveolar ridge-root; periodontal ligaments are hypothetically inhibiting this expansion. The short implants and transcrestal approach, if possible and predictable, minimize the risk of AAA injury. The prevention of bleeding is the most effective way to avoid complications.

##  Supplemental Information

10.7717/peerj.16439/supp-1 Supplemental Information 1 Raw data

## Additional Information and Declarations

### Data Availability

The following information was supplied regarding data availability: The raw data are available in a Supplementary File.
